# Dengue Expansion in Africa—Not Recognized or Not Happening?

**DOI:** 10.3201/eid2010.140487

**Published:** 2014-10

**Authors:** Thomas Jaenisch, Thomas Junghanss, Bridget Wills, Oliver J. Brady, Isabella Eckerle, Andrew Farlow, Simon I. Hay, Philip J. McCall, Jane P. Messina, Victor Ofula, Amadou A. Sall, Anavaj Sakuntabhai, Raman Velayudhan, G.R. William Wint, Herve Zeller, Harold S. Margolis, Osman Sankoh, the Dengue in Africa Study Group

**Affiliations:** Heidelberg University Hospital, Heidelberg, Germany (T. Jaenisch, T. Junghanss);; Oxford University Clinical Research Unit, Ho Chi Minh City, Vietnam (B. Wills);; University of Oxford, Oxford, UK (O.J. Brady, A. Farlow, S.I. Hay, J.P. Messina);; University of Bonn Medical Centre, Bonn, Germany (I. Eckerle);; Liverpool School of Tropical Medicine, Liverpool, UK (P.J. McCall);; US Army Medical Research Unit–Kenya, Nairobi, Kenya (V. Ofula);; Institut Pasteur, Dakar, Senegal (A.A. Sall);; Institut Pasteur, Paris, France (A. Sakuntabhai);; World Health Organization, Geneva, Switzerland (R. Velayudhan);; Environmental Research Group, Oxford (G.R.W. Wint);; European Centre for Disease Control and Prevention, Stockholm, Sweden (H. Zeller);; Centers for Disease Control and Prevention, San Juan, Puerto Rico, USA (H.S. Margoulis);; International Network for the Demographic Evaluation of Populations and Their Health, Accra, Ghana (O. Sankoh)

**Keywords:** dengue, dengue virus, viruses, epidemiology, disease incidence, disease burden, diagnostics, vector, policy, expansion, Africa

## Abstract

An expert conference on Dengue in Africa was held in Accra, Ghana, in February 2013 to consider key questions regarding the possible expansion of dengue in Africa. Four key action points were highlighted to advance our understanding of the epidemiology of dengue in Africa. First, dengue diagnostic tools must be made more widely available in the healthcare setting in Africa. Second, representative data need to be collected across Africa to uncover the true burden of dengue. Third, established networks should collaborate to produce these types of data. Fourth, policy needs to be informed so the necessary steps can be taken to provide dengue vector control and health services.

## Abstract 2

Addressing this expansion is essential before control and prevention of dengue are implemented.

Approximately 2.5–4 billion persons, 40%–60% of the world’s population, live in areas at risk for dengue virus (DENV) infection (*1*,*2*). With an estimated annual total of 390 million infections, dengue is the most frequent mosquito-borne viral disease worldwide (*3*). However, it is a neglected tropical disease (*4*), which might be the situation in Africa.

Since 1960, evidence of DENV transmission has been documented in 32 countries in Africa (*5*). The global share of apparent infections believed to occur in Africa (≈16% of 96 million infections worldwide) is of the same order of magnitude as that for Latin America (≈14%) (*3*). However, more limited occurrence data for Africa (*3*) and less consensus on the presence of DENV transmission in many countries in Africa (*2*) indicate that burden predictions remain uncertain.

With the recent growing global interest in dengue, the number of diagnostically robust reports from Africa has increased. All 4 DENV serotypes have now been documented to circulate in Africa, although DENV-2 has been reported most frequently (*6*). In 2010, DENV-3 outbreaks were reported in Tanzania, Zanzibar, the Comoros, Benin, and Cape Verde (*7*–*9*), followed by reports during 2011–2013 of substantial numbers of cases in Angola (*10*), Kenya (*11*), and Somalia (Centers for Disease Control and Prevention, unpub. data) caused by DENV-1. Preliminary phylogenetic studies indicate that the DENV-1 strain isolated in Angola clustered with strains from Côte d’Ivoire (1985, 1998, and 1999) and Nigeria (1968), which suggested transmission of endemic strains of DENV-1 in Africa (*12*). Case reports of returning travelers (*7*,*13*,*14*), and sequence data from sylvatic DENV strains (*15**,**16*) account for most of what is known at the genomic level regarding DENV circulating in Africa.

Unprecedented human mobility, rapid urban population growth, and large-scale changes in ecosystems have been associated with an increase in dengue transmission in Latin America and Asia, and are likely to favor spread of DENV into new locations (*17*–*20*). Reasons for an apparent emergence of DENV in Africa might include increased awareness of the disease, availability of better diagnostic tests, and improved access to specialized laboratory facilities. However, the range and lack of specificity of the symptoms of dengue indicate that clinical diagnosis is often difficult, even in dengue-endemic areas, where the index of suspicion is high among health care workers. Thus, hidden among the many febrile illnesses in Africa (malarial, bacterial, viral, rickettsial), dengue could have been overlooked, and the conspicuous mismatch between estimated disease incidence and actual number of reported cases might reflect a failure to consider dengue in the differential diagnosis of these common conditions.

In contrast, the clinical features of severe dengue are more distinct, although only a small fraction of DENV-infected patients progress to this stage (*21*). Until recently, severe dengue had been reported infrequently in Africa. However, among Ugandan peacekeepers deployed in Somalia, bleeding was reported in 12% of case-patients (Centers for Disease Control and Prevention, unpub. data). Six case-patients with severe dengue, including 1 who died (3% of 196 confirmed cases), were reported in an urban epidemic in Senegal in 2009 (*22*), and 11 deaths were reported in Luanda, Angola, in 2013 (*23*). Several possible reasons must be considered to explain why, if disease incidence across Africa is high, the severe end of the disease spectrum has not been more regularly detected. First, similar to the argument proposed earlier with respect to symptomatic dengue in general, clinicians unfamiliar with the disease may fail to consider the possibility of severe dengue in their differential diagnosis of patients with hypovolemic shock, severe bleeding, or severe organ dysfunction. Second, severe dengue occurs only rarely in Africa.

One hypothesis that has been proposed to support the rare occurrence of severe dengue in Africa suggests that protective genetic variants may be present in populations in Africa (*24*). The absence of recorded episodes of severe dengue in children in Haiti during 1994–1996, despite high transmission (*25*), and data for Cuba showing lower hospitalization rates for severe dengue among subpopulations of African ancestry (*26*–*29*), support this hypothesis. In addition, a study from Bahia, Brazil, assigned ancestry on the basis of genetic markers, and reported an association between African ancestry and a reduced risk for severe dengue (*30*). However, cases of severe dengue have been reported in the past from Africa (*31*–*33*), and illness and death from severe dengue have been documented during recent outbreaks when intensive surveillance was conducted.

An expert conference on Dengue in Africa was organized by the International Research Consortium on Dengue Risk Assessment, Management, and Surveillance (IDAMS) (European FP7; www.idams.eu) and the International Network for the Demographic Evaluation of Populations and Their Health (INDEPTH) (www.indepth-network.org) and held in Accra, Ghana, in February 2013 to consider key questions regarding dengue in Africa. Have we failed to observe an escalation of dengue in Africa causing a disease incidence of similar magnitude to that of Latin America, or is dengue truly not spreading into Africa for epidemiologic reasons that are not yet understood? The meeting addressed 4 major themes surrounding this central issue to advance our understanding of the epidemiology of dengue in Africa: 1) diagnostic tools and diagnostic capacity development; 2) improving estimates of the epidemiology and incidence of dengue; 3) biology of vectors and implications for vector control strategies; and 4) health policy, health services, and epidemic preparedness. The considerations and main suggestions for each theme are outlined in this report.

## Development of Diagnostic Tools and Diagnostic Capacity

Diagnostic capacity for dengue, as for virtually all causes of acute febrile illness (AFI), is limited in Africa (*34*). However, there are reliable serologic assays for the diagnosis of acute dengue, which require only modest technologic expertise and investment in infrastructure (*35*–*38*). Several rapid tests with reasonable sensitivity and specificity are available (*39*–*42*). Deployment of point-of-care testing or ELISA-based serologic assay methods (IgM, IgG, and nonstructural protein 1) at sentinel sites where risk for dengue is estimated to be high, would support accurate diagnosis and potentially raise awareness of the disease locally. A wide range of more sophisticated diagnostics, including PCR for DENV RNA, plaque-reduction neutralization tests, or microneutralization tests, are also available and could be integrated into national and regional reference laboratories (Figure), along with appropriate capacity development.

**Figure:**
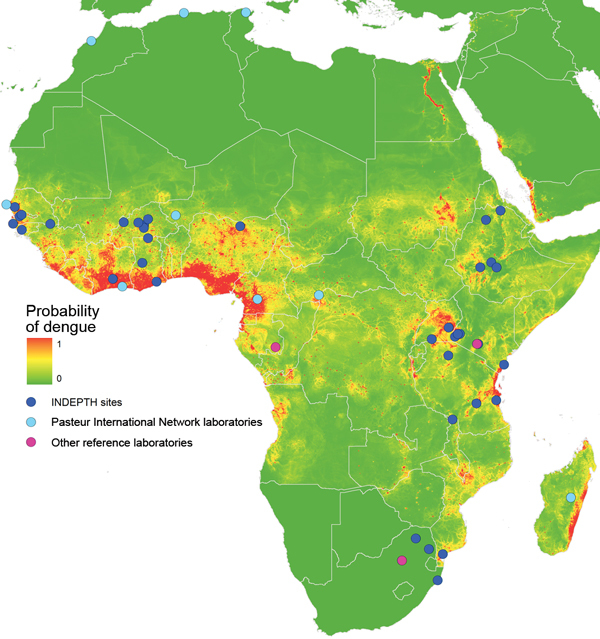
Dengue risk for Africa based on environmental niche modeling (*3*) and location of International Network for the Demographic Evaluation of Populations and Their Health (INDEPTH) member sites; Pasteur International Network Laboratories; and other reference laboratories (Centre International de Recherches Médicales de Franceville, Franceville, Gabon; Kenya Medical Research Institute/US Army Medical Research Unit, Nairobi, Kenya; and National Institute for Communicable Diseases, Johannesburg, South Africa).

Implementation of a tiered laboratory diagnostic system along these lines across Africa would facilitate unambiguous diagnosis of dengue infection in a subset of clinically suspected cases, provide quality assurance and quality control for feeder laboratories, and support a program of locally relevant scientific research. For example, 1 major step would be to evaluate these diagnostic tests in patients with AFI and take into account the high background endemicity of malaria and various co-circulating flaviviruses.

## Improving Estimates of Epidemiology and Burden of Dengue

Africa lacks systematic surveillance and reporting of many diseases, including dengue (*43*). Information from archived serum samples from a variety of repositories retrospectively tested for acute DENV infection could be used to clarify the extent of transmission across Africa. This information includes studies of AFI, malaria, influenza-like illness, yellow fever, or measles-negative specimens in rash–illness studies. Prospective AFI studies are also needed to ascertain whether case definitions for dengue in Africa differ from those for Asia, the Americas, and Oceania.

The African INDEPTH member sites provide a unique opportunity to conduct denominator-based research for more reliable quantification of the burden of dengue in Africa. The INDEPTH network includes 35 health and demographic surveillance systems (HDSSs) in Africa, which are producing longitudinal demographic data about lives of persons in low-income and middle-income countries (*44*). Prospective efforts to quantify the presence of dengue can be guided by existing reports, as well as by published risk maps (*3*). Analysis of climatic, demographic, and socioeconomic variables of precise locations where dengue has been reported in Africa enables generation of predictive models that can suggest other environments suitable for DENV transmission (Figure). These models can provide a basis for future hypothesis-driven research regarding the presence and quantification of dengue in Africa. Results of such research would, in return, improve the accuracy of subsequent risk maps. In addition, because molecular epidemiologic studies and phylogenetic analysis of DENV strains will provide more informed insight into origins and distribution of DENV across Africa, submission of sequence data for as many samples as possible should also be encouraged.

## Biology of Vectors and Implications for Vector Control Strategy

Should dengue be identified as imposing a major burden in Africa, it will be essential to understand the biology and behavior of local vectors because these factors will influence transmission, as well as selection and design of effective control tools and strategies. The 2 major DENV vectors (*Aedes aegypti* and *Ae. albopictus* mosquitoes*)* are present in Africa. In Senegal, other *Aedes* mosquito species have also been shown to be involved in transmission of sylvatic dengue (*45*).

At least 2 forms of *Ae. aegypti* mosquitoes need to be distinguished in Africa: an ancestral forest or sylvan form, and a global domestic form. These 2 forms differ in distribution, behavior (*46*,*47*), and potentially in vector competence for DENV (*48*). Additional genetic variability might be present within the global domestic form (*49*). Older studies have reported unusual bionomics, such as high levels of nocturnal bloodfeeding, exophily, or dispersal in *Ae. aegypti* mosquitoes in Africa (*50*,*51*).

*Ae. albopictus* mosquitoes continue to spread through Africa since they were first reported in Nigeria in 1991, and these mosquitoes might play a major role in the epidemiology of dengue in Africa (*52*). This vector was incriminated as the sole vector in a dengue outbreak in central Africa in 2007 (*20*). Recent studies in Cameroon reported *Ae. albopictus* mosquitoes blood-feeding on humans (*53*) and breeding in domestic water containers (*54*) at levels that exceed those seen outside Africa.

Dengue vector control requires thorough knowledge of vector biology and behavior to be effective (*55*). The effect of existing malaria vector control programs (targeted for anopheline species of mosquitoes) on DENV vectors is likely to be minimal because malaria control in Africa is typically conducted in rural settings, targeting the nocturnally active vectors inside homes with long-lasting insecticide-treated bed nets or in the large natural water bodies where breeding occurs. Conversely, *Aedes* spp. mosquitoes are diurnally active, which indicates that long-lasting insecticide-treated bed nets are ineffective for dengue vector control (*17*,*55*,*56*). These mosquitoes also breed in artificial containers in urban domestic environments, which makes management of rural water bodies equally irrelevant for dengue control. Spraying of insecticides in and around homes is a common approach for limiting dengue vector populations, but studies have not shown it to be an effective control strategy on its own (*57*).

## Health Policy, Health Services, and Epidemic Preparedness

Given a lack of awareness of dengue in Africa, it is perhaps not surprising that dengue control is a low-priority health policy in this region (*3*). DENV transmission in Asia and Latin America is concentrated around unplanned urban and semi-urban areas with poor infrastructure (*18*), a condition that is widespread and increasing in Africa (*58**,**59*). Dengue epidemics in densely populated areas regularly drive health facilities in Asia and Latin America to their limits (*60*). Therefore, health services in Africa should be prepared to face similar challenges, and this preparation relies crucially on collection of more and improved baseline epidemiologic data. These data are essential for monitoring trends of disease occurrence, identifying epidemic-prone areas, and assessing the potential benefits of a future dengue vaccine on burden reduction in the region (*61*).

In summary, the expert conference in Accra highlighted 4 key action points. First, dengue diagnostic tools must be made more widely available in the health care setting in Africa. Second, representative data need to be collected across Africa to uncover the true incidence of dengue and more clearly define its transmission in the region. Third, established networks, such as African laboratory networks, the Pasteur International network, the INDEPTH network, and others, should collaborate to produce these needed types of data. Fourth, policy needs to be informed by improved information to take necessary steps for dengue vector control and provision of health services. Addressing these issues will be essential before any major future decisions regarding control or prevention of dengue in Africa can be made.
